# Increased *LGALS3* expression independently predicts shorter overall survival in patients with the proneural subtype of glioblastoma

**DOI:** 10.1002/cam4.2075

**Published:** 2019-03-07

**Authors:** Xia He, Sunfu Zhang, Junchen Chen, Dekang Li

**Affiliations:** ^1^ Department of Integrated Traditional Chinese and Western Medicine Sichuan Bayi Rehabilitation Center/Sichuan Provincial Rehabilitation Hospital Chengdu China; ^2^ Department of Neurosurgery The First People's Hospital of Yibin Yibin China; ^3^ Department of Neurosurgery Sichuan Bayi Rehabilitation Center/Sichuan Provincial Rehabilitation Hospital Chengdu China

**Keywords:** glioblastoma, *LGALS3*, LGALS3BP, proneural subtype

## Abstract

In the current study, we tried to study the expression of *LGALS3 *and *LGALS3BP*, their potential as prognostic markers and the possible genetic/epigenetic mechanisms underlying their dysregulation in different subtypes of glioblastoma (GBM). An in silico retrospective study was performed using large online databases. Results showed that *LGALS3* and *LGALS3BP* were upregulated at both RNA and protein levels in GBM tissue and were generally associated with shorter overall survival (OS) in GBM patients. However, in subgroup analysis, we only found the association in proneural subtype. The copy number alterations did not necessarily lead to *LGALS3/LGALS3BP* dysregulation. In the proneural subtype of GBM patients, hypermethylation of the two CpG sites (cg19099850 and cg17403875) was associated with significantly lower expression of *LGALS3*. In univariate and multivariate analysis, *LGALS3 *expression independently predicted shorter OS in the proneural subtype of GBM (HR: 1.487, 95% CI: 1.229‐1.798, *P* < 0.001), after adjustment of age, gender, *IDH1* mutations, temozolomide chemotherapy, radiotherapy and *LGALS3BP* expression. In comparison, *LGALS3BP* lost the prognostic value in multivariate analysis. Based on these findings, we infer that *LGALS3* expression serves as an independent biomarker of shorter OS in the proneural subtype of GBM, the expression of which might be regulated in an epigenetic manner.

## 1 INTRODUCTION

Glioblastoma multiforme (GBM) (WHO grade IV Astrocytoma), is the most malignant tumor that begins within the brain. It accounts for about 15% of all brain tumors, with overall median survival only around 15 months.^1^ In the past decades, two prognostic biomarkers attracting significant interest were isocitrate dehydrogenase (*IDH*) mutations and O(6)‐methylguanine‐DNA methyltransferase (*MGMT*) promoter methylation.^2^

*IDH* mutations lead to the loss of native enzymatic activity and subsequent abnormal production of 2‐hydroxyglutarate (2‐HG).^3^ 2‐HG can inhibit the enzymic activity of DNA demethylases and result in increased DNA methylation. Due to this influence, a subset of GBM shows Glioma CpG Island Methylation Phenotype (G‐CIMP).^4^ GBM patients with *IDH1* mutations or those belonging to the G‐CIMP phenotype have significantly improved OS.^5^ Besides, *MGMT* promoter methylation is also a powerful and independent indicator of therapeutic responses among GBM patients who received chemotherapy with alkylating agents.^6^ However, these markers have their own limitations in clinical use. *IDH1* mutations are rare in primary GBM cases.^7^ In comparison, the prognosis value of *MGMT *promoter might depend on the chemotherapeutic substances used 6 and might lose the value under some conditions.^8^, ^9^ Therefore, it is quite meaningful to further explore other potential prognostic markers in GBM. However, GBM is a not a homogenous disease, but has heterogeneous histological features.^10^, ^11^ The Cancer Genome Atlas (TCGA) researchers divided GBM into four subtypes (Proneural, Neural, Classical and Mesenchymal) according to their gene expression pattern.^12^ Besides the genomic differences, these subtypes varied significantly in survival length, patient age and treatment response. Therefore, exploring specific prognostic marker of the GBM subtypes might support better therapeutic management.

The galectins are a group of beta‐galactoside–binding proteins that involve in regulating cell‐cell and cell‐matrix interactions.^13^ Till now, galectin‐1, ‐2, ‐3, ‐4, ‐7, ‐8, ‐9, ‐10 and ‐12 have been identified in humans. Galectin‐3 encoded by *LGALS3* gene plays an important role in cell adhesion, growth, differentiation, apoptosis, as well as angiogenesis in both normal and cancerous tissues.^14^ Its upregulation might serve as a valuable prognostic marker in multiple cancers, such as breast cancer,^15^ gastric cancer,^16^, ^17^ colorectal cancer^18^ and liver cancer.^19^ Upregulated *LGALS3* was also observed in GBM tissues.^20^, ^21^ Galectin‐3–binding protein (Gal‐3BP) is a secreted glycoprotein encoded by *LGALS3BP* gene. In the microenvironment of human neuroblastoma, Gal‐3BP interacts with Galectin‐3 (Gal‐3) in bone marrow mesenchymal stem cells (BMMSC) and induces transcriptional upregulation of IL‐6, via the Gal‐3BP/Gal‐3/Ras/MEK/ERK signaling pathway.^22^, ^23^ These findings suggest that these two genes may collaboratively participate in the pathological process of cancer.

In the current study, we tried to study the expression of *LGALS3 *and *LGALS3BP*, their potential as prognostic markers and the possible genetic/epigenetic mechanism underlying their dysregulation in different subtypes of glioblastoma (GBM), by using large online databases.

## 2 PATIENTS AND METHODS

### 2.1 Retrospective analysis using data from TCGA‐GBM

The level‐3 data in TCGA‐GBM were obtained using the UCSC Xena Browser (https://xenabrowser.net/heatmap/).^24^ The recurrent tumors and cases that had a history of neoadjuvant treatment were excluded. Affymetrix Human Genome U133 Array Strip (AffyU133a) data was used to quantify gene expression. Based on these criteria, a total of 508 primary tumor cases and 10 adjacent normal tissue cases were included in this study. The genomic, clinicopathological and survival data of the included patients were downloaded. Briefly, the data included *IDH1* mutations, gene expression subtypes, age at initial pathologic diagnosis, longest tumor dimension, gender, karnofsky performance score (KPS), overall survival (OS) time, temozolomide chemotherapy status, radiation therapy status, *LGALS3/LGALS3BP* expression, *LGALS3/LGALS3BP *DNA copy number alterations (CNAs, calculated by an algorithm called Genomic Identification of Significant Targets in Cancer 2.0 [GISTIC2])^25^ and their DNA methylation (quantified by Infinium HumanMethylation27 BeadChip). In the GISTIC2, CNAs were defined as −2: homozygous deletion; −1: heterozygous deletion, 0: copy‐neutral, +1: low–level copy gain, +2: high–level amplification.

MGMT‐STP2 model,^26^ which includes two CpG sites (cg12434587 and cg12981137) was used determine the *MGMT* promoter methylation status.

### 2.2 Data mining in the R2 web‐based application

The association between *LGALS3/LGALS3BP* expression and OS in GBM patients was also analyzed using genomic and survival data in GSE16011 from GEO datasets,^27^ using the R2 web–based application (https://hgserver1.amc.nl/cgi-bin/r2/main.cgi). Only the GBM cases in GSE16011 were included in Kaplan‐Meier survival analysis. The best cutoff was identified using scan model.

### 2.3 Data mining in the HPA

*LGALS3* and *LGALS3BP* protein expression in normal brain tissues (typically cerebral cortex and hippocampus) and GBM tissues was examined using data from the Human Protein Atlas (HPA) (available fromwww.proteinatlas.org).^28^, ^29^ Immunochemical images and protein scoring data of *LGALS3* and *LGALS3BP* were retrieved.

In this database, protein expression score is based on the combination of staining intensity (negative, weak, moderate or strong) and fractions (<25%, 25%‐75% or >75%) of the immunohistochemical images. Protein expression score is defined as: not detected (negative or weak <25%); low (weak combined with either 25%‐75% or 75%); medium (moderate <25%—low; moderate combined with either 25%‐75% or 75%); and high (strong <25%—medium, strong combined with either 25%‐75% or 75%). Besides, protein expression scores are manually adjusted as necessary when evaluated by the expert annotators.^28^, ^29^

### 2.4 Statistical analysis

Statistical analyses were conducted using SPSS 25.0 software (SPSS, Chicago, IL) and GraphPad Prism 7.04 (GraphPad Inc, La Jolla, CA). One‐way ANOVA with Bonferroni post hoc tests and Welch's *t* test (unequal variances *t *test) were performed to assess the statistical differences. Kaplan‐Meier OS curves were generated using GraphPad Prism 7.04. Receiver operating characteristic (ROC) analysis for death detection was performed to identify the best cutoff (Youden index) for gene expression in Kaplan‐Meier curves. The difference between the curves was compared using the Log–rank test. The independent prognostic value of *LGALS3/LGALS3BP* expression in proneural subtype was assessed using the univariate and multivariate Cox regression models. *P* < 0.05 was considered as statistically significant.

## 3 RESULTS

### 3.1 Both *LGALS3* and *LGALS3BP *RNA expression were upregulated in GBM tissues compared with adjacent normal tissues

**Figure 1:**
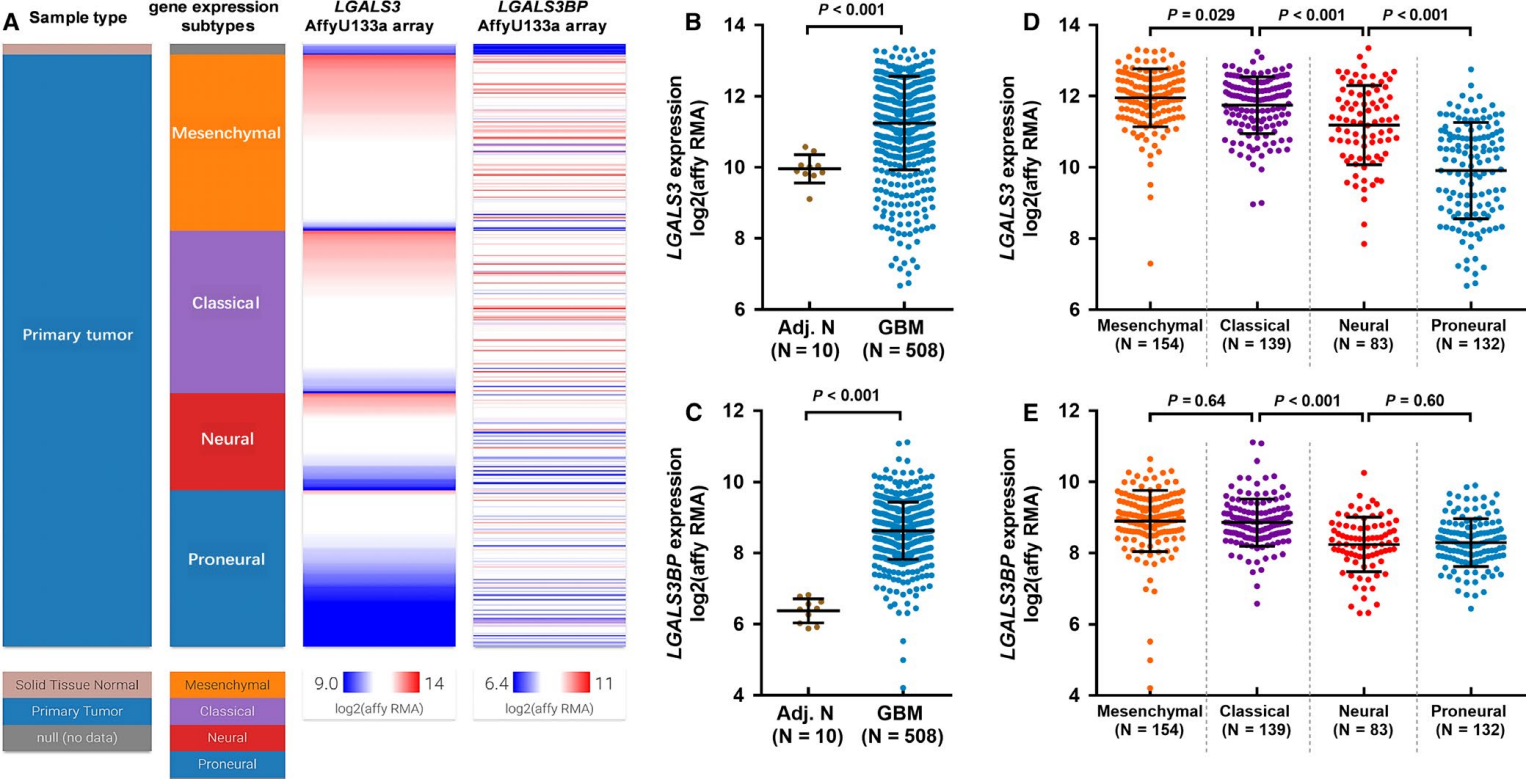
The expression profile of *LGALS3* and *LGALS3BP *in glioblastoma (GBM) tissues and the adjacent normal tissues. A, Heatmap showing the expression of *LGALS3* and *LGALS3BP *in GBM tissues and the adjacent normal tissues. B‐E, Plot charts comparing the expression of *LGALS3* (B and D) and *LGALS3BP *(C and E) between GBM tissues and the adjacent normal tissues (B‐C) and among the four subtypes of GBM (D‐E)

In TCGA‐GBM, 508 cases of primary GBM and 10 adjacent normal tissues were subjected to AffyU133a microarray analysis of gene expression. Using the array data, we compared the expression of *LGALS3* and *LGALS3BP *in GBM and the adjacent normal tissues, as well as the different subtypes of GBM (Figure 1A). Results showed that both *LGALS3* and *LGALS3BP *were significantly upregulated in GBM tissues compared with adjacent normal tissues (Figure 1A‐C). Since the distinct molecular subtypes of GBM show varying prognosis and responses to aggressive chemotherapy and radiotherapy, we also examined the expression profiles of these two genes in the subtypes. Group comparison showed that *LGALS3 *expression varied significantly among the four subtypes, in which the mesenchymal and proneural subtypes had the highest and lowest expression, respectively (Figure 1D). In comparison, *LGALS3 *expression in the neural and proneural subtypes was significantly lower than that in the mesenchymal and classical subtypes (Figure 1E).

### 3.2 *LGALS3* and *LGALS3BP* protein expression was not detectable in glial cells in normal brain tissues, but was detectable in GBM tissues

**Figure 2:**
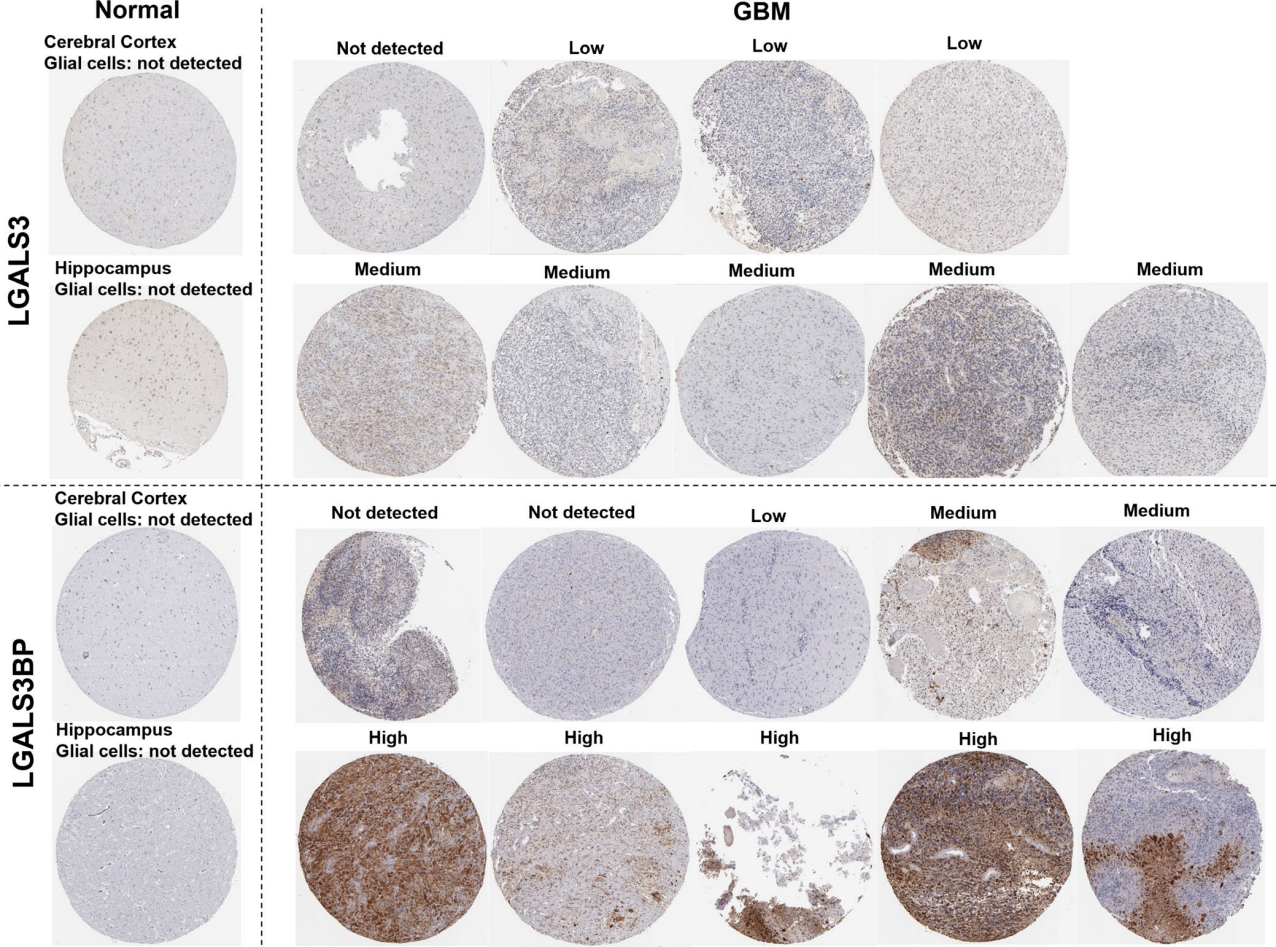
LGALS3 and LGALS3BP protein expression in normal brain tissues and in glioblastoma (GBM) tissues. IHC staining of LGALS3 (up) and LGALS3BP (down) protein expression in normal brain tissues (left, including cerebral cortex and hippocampus) and in GBM tissues. Images credit: The Human Protein Atlas. Images and protein scoring were obtained from: v18.proteinatlas.org, via: https://www.proteinatlas.org/ENSG00000131981-LGALS3/tissue/cerebral+cortex; https://www.proteinatlas.org/ENSG00000131981-LGALS3/tissue/hippocampus; httpsihc://www.proteinatlas.org/ENSG00000131981-LGALS3/pathology/tissue/glioma#ihc; https://www.proteinatlas.org/ENSG00000108679-LGALS3BP/tissue/cerebral+cortex; https://www.proteinatlas.org/ENSG00000108679-LGALS3BP/tissue/hippocampus and https://www.proteinatlas.org/ENSG00000108679-LGALS3BP/pathology/tissue/glioma#ihc

Using IHC staining images and protein expression scoring in the HPA, we examined LGALS3 and LGALS3BP protein expression in normal brain and GBM tissues. According to the data in the HPA, LGALS3 and LGALS3BP protein expression was not detectable in glial cells in normal brain tissues (Figure 2, left). In comparison, among 9 cases of GBM with LGALS3 examined, 8 cases showed positive LGALS3 staining (3 low and 5 medium) (Figure 2, right). In addition, 8 out of 10 GBM cases had positive LGALS3BP staining (1 low, 2 medium and 5 high). These findings confirmed that LGALS3 and LGALS3BP were expressed at the protein level in GBM tissues.

### 3.3 *LGALS3 *and *LGALS3BP* upregulation was associated with unfavorable OS in GBM patients

**Figure 3:**
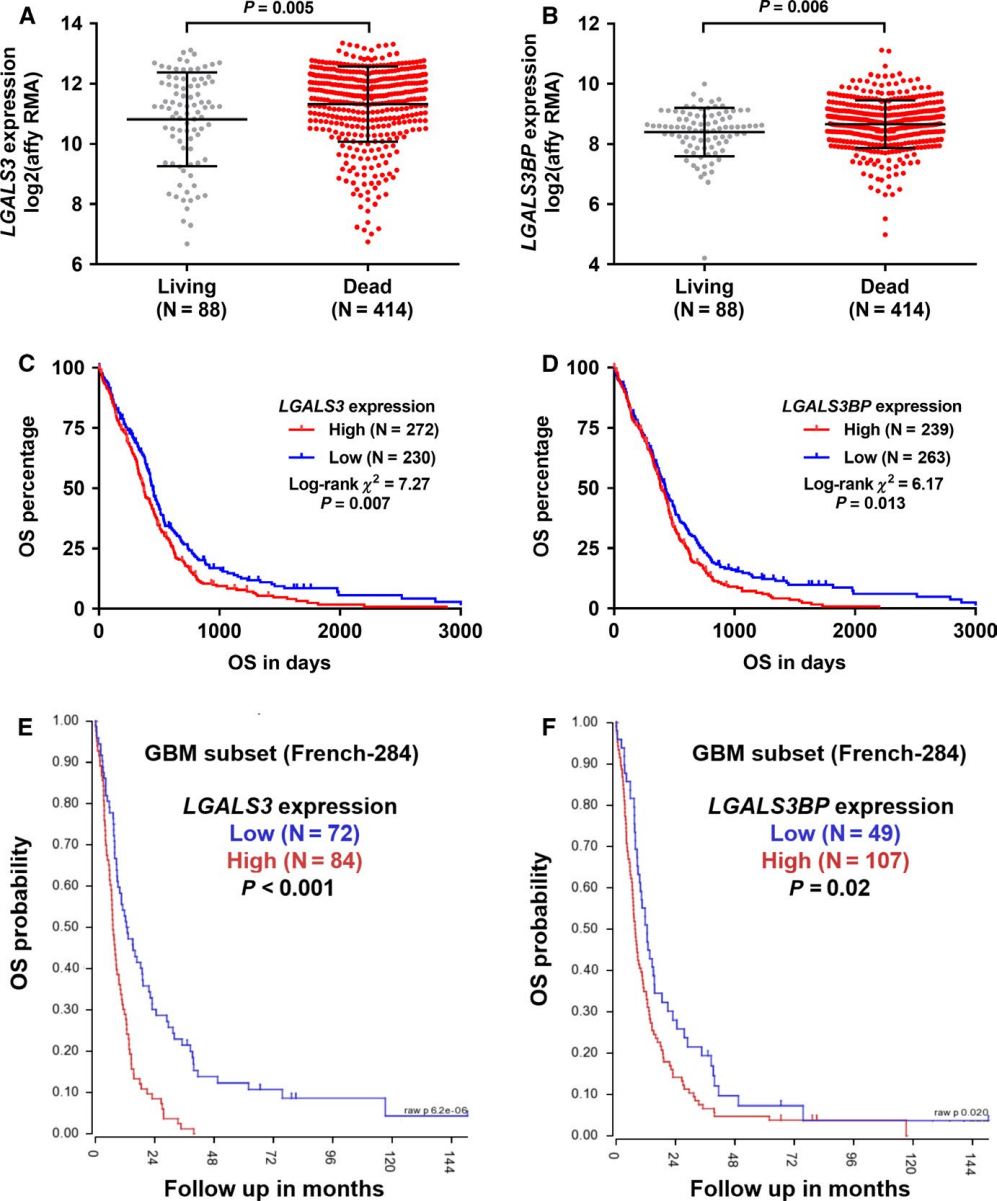
*LGALS3/LGALS3BP* expression and overall survival (OS) of glioblastoma (GBM). A and B, Plot charts comparing *LGALS3 *(A)*/LGALS3BP* (B) expression between the living and deceased GBM cases in TCGA‐GBM. C and D, Kaplan‐Meier curves of OS in GBM patients in TCGA‐GBM. Patients were separated into two groups according to the best cutoff of *LGALS3 *(C)*/LGALS3BP* (D) expression identified in the ROC analysis for death detection. E and F, Kaplan‐Meier curves of OS in GBM patients in GSE16011 from GEO datasets.^27^ Patients were separated into two groups according to the best cutoff of *LGALS3 *(E)*/LGALS3BP* (F) expression

By comparing *LGALS3 *and *LGALS3BP *expression between the deceased and living GBM cases, we found that the deceased group had significantly elevated *LGALS3 *and *LGALS3BP *expression (*P* = 0.005 and *P* = 0.006, respectively) (Figure 3A,B). Then, we tried to explore the association between the expression of these two genes and the survival outcomes by generating Kaplan‐Meier curves, using the survival data in TCGA‐GBM. Via setting the best cutoff identified in the ROC analysis for death detection, we found that the high *LGALS3 *expression group and the high *LGALS3BP *expression group had significantly shorter OS compared to the respective low expression group (*P* = 0.007 and *P* = 0.013, respectively) (Figure 3C,D). To verify these trends, we also used genomic and survival data in another study (Tumor Glioma French database, GSE16011 from GEO datasets 27), which included 156 cases of GBM out of 284 glioma cases. Results confirmed the association between the high gene expression and unfavorable OS in GBM patients (Figure 3E,F).

### 3.4 Kaplan‐Meier OS analysis in the four molecular subtypes of GBM

**Figure 4:**
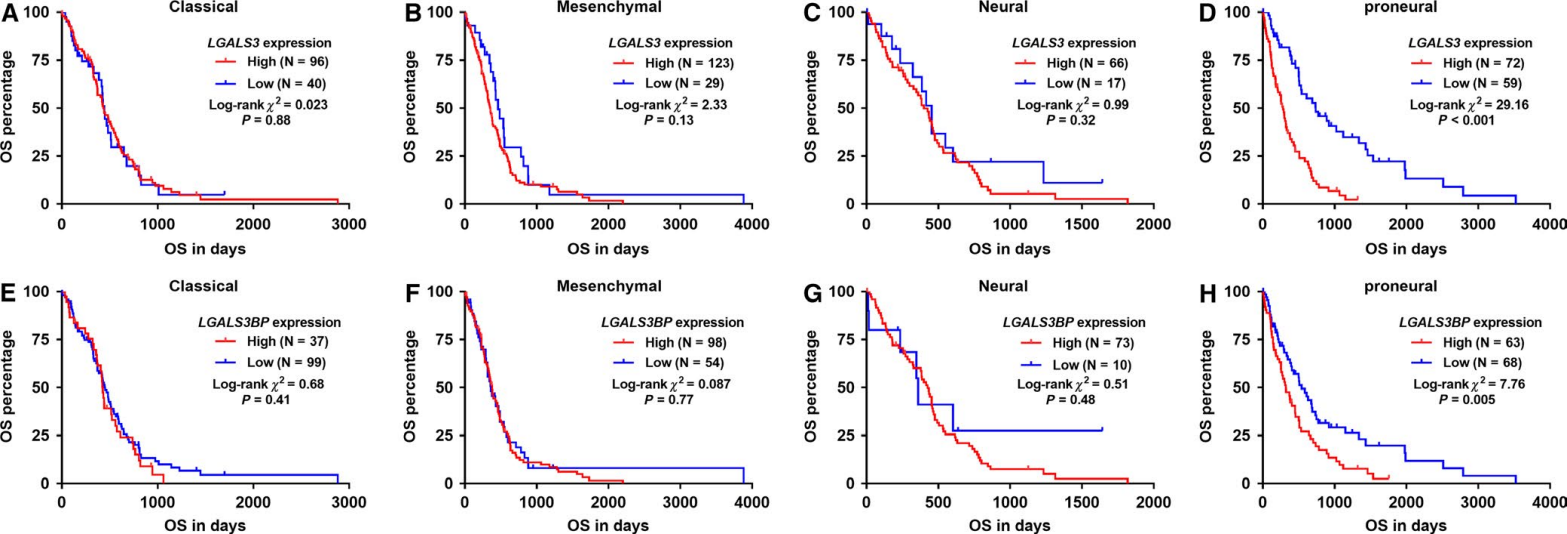
*LGALS3/LGALS3BP* expression and overall survival (OS) of glioblastoma (GBM) subtypes. A‐H, Kaplan‐Meier curves of OS in the 4 molecular subtypes of GBM patients in TCGA‐GBM. Patients were separated into two groups according to the best cutoff of *LGALS3 *(A‐D)*/LGALS3BP* (E‐H) expression identified in the ROC analysis of death detection

Since we found that both *LGALS3 *and *LGALS3BP *expression varied significantly in the four subtypes of GBM, we then tried to explore whether the association between the high gene expression and unfavorable survival was consistent in the four subtypes. Subgroup analysis suggested that *LGALS3 *and *LGALS3BP *expression had no prognostic value in terms of OS in mesenchymal, classical and neural subtypes (Figure 4A,C,E,G). In comparison, the associations were confirmed in proneural subtype (Figure 4D,H).

### 3.5 *LGALS3 *expression was not correlated with its DNA CNAs, but was associated with *IDH1* mutations in proneural GBM

**Figure 5:**
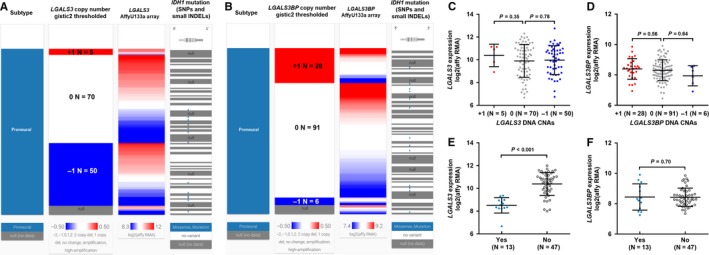
The correlation between *LGALS3/LGALS3BP* expression and their DNA CNAs and *IDH1* mutations in proneural glioblastoma (GBM). A and B, Heatmaps showing the correlation between *LGALS3*(A)*/LGALS3BP*(B) expression and their DNA CNAs and *IDH1* mutations in proneural subtype. C and D, Plot charts comparing *LGALS3*(C)*/LGALS3BP*(D) in groups with different DNA CNAs. E and F, Plot charts comparing *LGALS3*(E)*/LGALS3BP*(F) in groups with or without *IDH1* mutations

Using genomic data in TCGA‐GBM, we further investigated the potential genomic and epigenetic alterations associated with dysregulation of these two genes. The proneural subtype was prone to have*LGALS3* heterozygous deletion (50/125, 40%) (Figure 5A). In comparison, *LGALS3BP* amplification (28/125, 22.4%) was more common than heterozygous deletion (6/125, 4.8%) (Figure 5B). However, these genetic alterations did not necessarily lead to *LGALS3/LGALS3BP* dysregulation (Figure 5C‐D). Previous studies found that *IDH1* mutation is an important prognostic marker in GBM patients and is associated with hypermethylation status of a series of genes.^12^, ^27^ By checking the correlation between these two genes and *IDH1* mutations, we found that the *IDH1* mutation group had significantly lower *LGALS3* expression (*P* < 0.001, Figure 5E). But this association was not observed in *LGALS3BP* expression (*P* = 0.70, Figure 5F).

### 3.6 *LGALS3* expression is related to its DNA methylation status in proneural GBM

**Figure 6:**
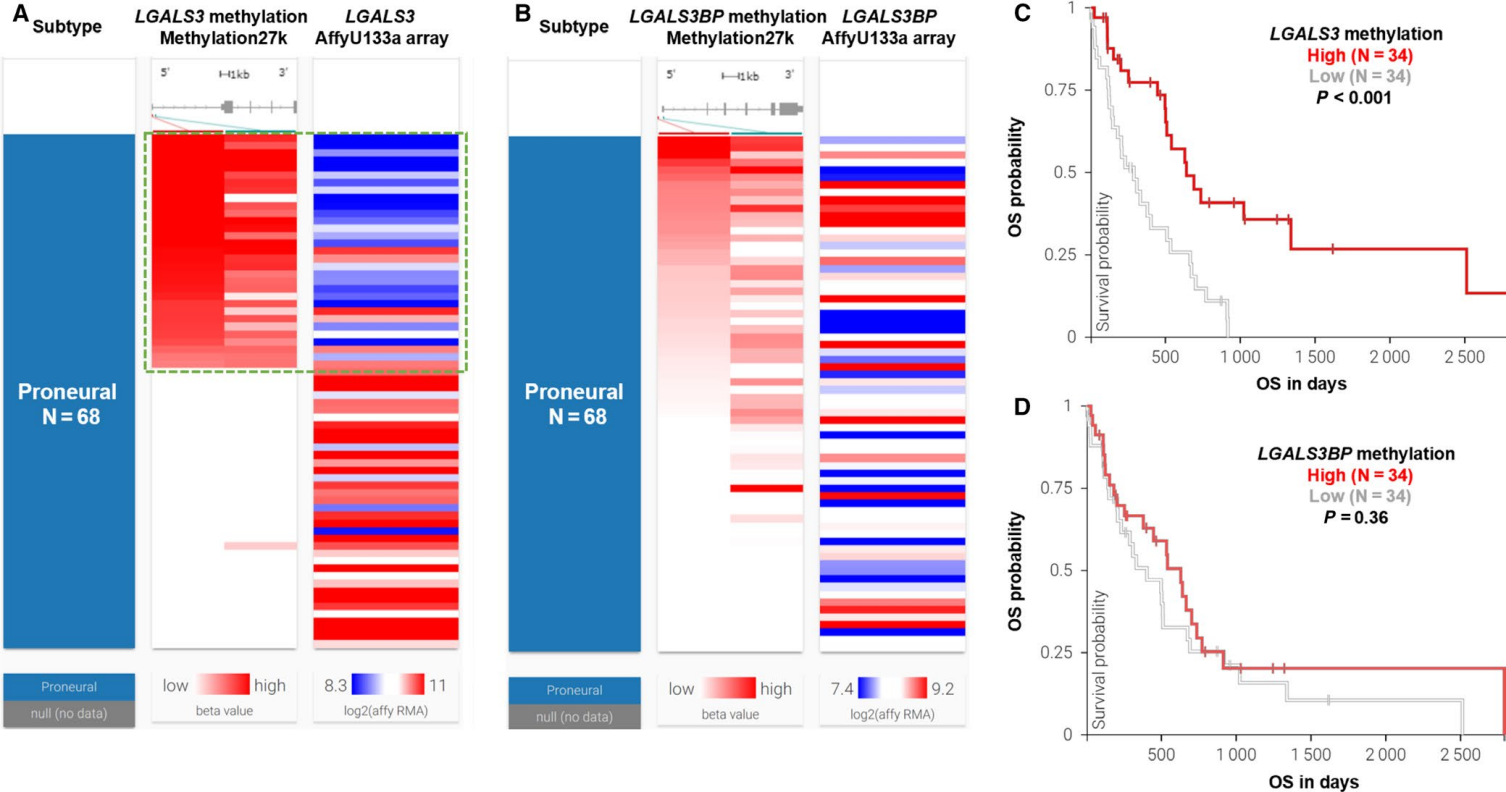
The correlation between *LGALS3/LGALS3BP* expression and their DNA methylations in proneural glioblastoma (GBM). A and B, Heatmaps showing the correlation between *LGALS3*(A)*/LGALS3BP*(B) expression and their DNA methylations in proneural subtype. C and D, Plot charts comparing *LGALS3*(C)*/LGALS3BP*(D) in groups with different DNA CNAs. E and F, Kaplan‐Meier curves of overall survival (OS) in the proneural subtype of GBM patients in TCGA‐GBM. Patients were separated into two groups according to the median methylation level of the two CpG sites of *LGALS3*(E)*/LGALS3BP*(F) measured in methylation 27k

Since *IDH1* mutations are generally associated with hypermethylation status, we then checked whether *LGALS3* expression was related to its DNA methylation level. By examining the correlation between *LGALS3* expression and its DNA methylation (methylation 27k data), we found that the group of patients with hypermethylation of the two CpG sites (cg19099850 and cg17403875) had significantly lower expression of *LGALS3* (Figure 6A, green box). In comparison, this association was not observed in terms of *LGALS3BP* expression (Figure 6B). By separating the patients according to median *LGALS3/LGALS3BP* methylation, we found that the *LGALS3* hypermethylation group had significantly better OS (Figure 6C). No significant association was observed between *LGALS3BP* methylation and OS (Figure 6D).

### 3.7 *LGALS3* expression was an independent prognostic indicator of OS in proneural subtype, after the adjustment of *IDH1* mutations

To explore the independent prognostic value of *LGALS3* expression in terms of OS in proneural subtype, we performed univariate and multivariate analysis based on COX regression model. The clinicopathological, genomic and survival data used were given in supplementary Table S1. Univariate analysis showed that older age, male patients, no *IDH1* mutations, no temozolomide chemotherapy, no radiotherapy, increased *LGALS3* expression and increased *LGALS3BP* expression were risk factors of shorter OS (Table 1). In multivariate analysis, increased *LGALS3* expression was independently associated with shorter OS (HR: 1.487, 95% CI: 1.229‐1.798, *P* < 0.001) (Table 1), after adjustment of other risk factors. However, *LGALS3BP* expression had no independent prognostic value (Table 1).

**Table 1:** Univariate and multivariate analysis of overall survival (OS) in the proneural subtype of glioblastoma (GBM)

Parameters	Univariate analysis				Multivariate analysis			
	*P*	HR	95% CI (lower/upper)		*P*	HR	95% CI (lower/upper)	
Age (continuous)	**<0.001**	1.038	1.024	1.053	**0.034**	1.018	1.001	1.035
Longest tumor dimension (cm, continuous)	0.993	1.003	0.579	1.737				
Gender								
Male (N = 79)		1.000						
Female (N = 52)	**0.044**	0.657	0.436	0.989	**0.008**	0.521	0.322	0.843
KPS								
>80 (N = 12)		1.000						
≤80 (N = 89)	0.062	2.105	0.964	4.595				
*IDH1* mutations								
Yes (N = 13)		1.000						
No (N = 47)	**0.004**	3.930	1.541	10.020	0.259	1.868	0.631	5.525
Temozolomide chemotherapy								
Yes (N = 67)		1.000						
No (N = 55)	**0.010**	1.718	1.141	2.586	0.918	1.025	0.644	1.632
Radiation therapy								
Yes (N = 97)		1.000						
No (N = 25)	**<0.001**	4.803	2.895	7.967	**<0.001**	5.460	2.964	10.056
*MGMT *promoter methylation	0.767	0.841	0.268	2.641				
*LGALS3 *expression	**<0.001**	1.640	1.383	1.946	**<0.001**	1.487	1.229	1.798
*LGALS3BP *expression	**0.005**	1.450	1.118	1.881	0.395	1.164	0.820	1.652

Bold indicates *P* < 0.05

## 4 DISCUSSION

A series of studies confirmed the oncogenic properties of *LGALS3* expression in glioma. Galectin‐3 expression is correlated with the malignant potential of tumors in the central nervous system.^30^ Its expression could be induced under hypoxic and nutrient deprived microenvironments, which protects cells from cell death.^20^ In addition, it also enhances GBM cell motility.^31^ However, previous studies found that *LGALS3* expression varied significantly in different types of glioma, such as supratentorial pilocytic astrocytoma, astrocytoma, anaplastic astrocytoma and glioblastoma.^21^, ^32^ These findings suggest that Galectin‐3 might have varying regulatory effects on different types of glioma. In terms of *LGALS3BP*, it is characterized as an onco‐protein that regulates the malignant behaviors of multiple cancers.^33^, ^34^, ^35^ In this study, we confirmed the upregulation of *LGALS3* and *LGALS3BP* at both RNA and protein level in GBM tissues. Besides, we found that *LGALS3* expression was generally associated with shorter OS in GBM patients. Since we observed that these two genes had different expression in the 4 subtypes of GBM, we further performed subtype group analysis to check the robustness of the finding. In subgroup analysis, we only found the association between *LGALS3*/*LGALS3BP *expression and unfavorable OS in proneural subtype, but not in other subtypes.

Among the subtypes, the proneural subtype has the most frequent mutations in the*IDH1* gene. In addition, the proneural subtype had the longest median OS, around 17 months.^36^ In this study, we found that among 60 cases of proneural subtype patients with *IDH1* mutations examined, only 13 cases had mutations. Therefore, although IDH1 mutations might be a powerful prognostic marker, it is still necessary to explore other robust prognostic biomarkers for the large proportion of proneural patients without *IDH1* mutations. In univariate analysis, we found that the promoter methylation status of *MGMT* might not be a prognostic marker in proneural subtype, when not considering the therapeutic strategy. This finding is consistent with previous studies that suggest the prognostic value of MGMT promoter methylation dependents on the chemotherapeutic substances used.^6^ By conducting univariate and multivariate analysis, we confirmed that *LGALS3 *expression was an independent prognostic indicator of shorter OS in the proneural subtype of GBM (HR: 1.487, 95% CI: 1.229‐1.798, *P* < 0.001), after the adjustment of age, gender, *IDH1* mutations, temozolomide chemotherapy, radiotherapy and *LGALS3BP* expression. In comparison, although *LGALS3BP* expression is a risk factor in univariate analysis, it lost the prognostic value in multivariate analysis. These findings suggest that *LGALS3* expression might be a valuable prognostic biomarker in the proneural subtype of GBM.

*LGALS3 *methylation expression is associated with the loss of the galectin‐3 expression in the mucinous colorectal carcinomas,^37^ prostate cancer^38^ and breast cancer,^39^ suggesting that its expression is controlled in an epigenetic manner. Since *IDH1* mutations lead to hypermethylation status and G‐CIMP phenotypes^4^ and is associated with suppressed *LGALS3* expression, we examined the correlation between the methylation status of *LGALS3* and their expression. Results showed *LGALS3 *DNA hypermethylation was associated with decreased *LGALS3 *expression. This phenomenon suggests that DNA methylation is a potential epigenetic alteration leading to *LGALS3* dysregulation in the proneural subtype of GBM.

This study also had some limitations. Firstly, some clinical information, such as surgical resection and the extent of resection were not recorded in TCGA‐GBM. This might impair the credibility of the findings. Secondly, although the Tumor Glioma French database was used to validate the prognostic value of *LGALS3*, this database had no information of molecular subtypes. Therefore, more detailed validation analysis based on a large cohort of the proneural subtype is required in the future.

## 5 CONCLUSION

*LGALS3* expression serves as an independent biomarker of shorter OS in the proneural subtype of GBM, the expression of which might be regulated in an epigenetic manner.

## CONFLICT OF INTEREST

The authors declare no potential conflict of interest.

## References

[1] Martinez R , Rohde V , Schackert G . Different molecular patterns in glioblastoma multiforme subtypes upon recurrence. J Neurooncol. 2010;96(3):321‐329. 19644652 10.1007/s11060-009-9967-4 PMC2811648

[2] Ng K , Kim R , Kesari S , Carter B , Chen CC . Genomic profiling of glioblastoma: convergence of fundamental biologic tenets and novel insights. J Neurooncol. 2012;107(1):1‐12. 22002595 10.1007/s11060-011-0714-2

[3] Turkalp Z , Karamchandani J , Das S . IDH mutation in glioma: new insights and promises for the future. JAMA Neurol. 2014;71(10):1319‐1325. 25155243 10.1001/jamaneurol.2014.1205

[4] Noushmehr H , Weisenberger DJ , Diefes K , et al. Identification of a CpG island methylator phenotype that defines a distinct subgroup of glioma. Cancer Cell. 2010;17(5):510‐522. 20399149 10.1016/j.ccr.2010.03.017 PMC2872684

[5] Guan X , Vengoechea J , Zheng S , et al. Molecular subtypes of glioblastoma are relevant to lower grade glioma. PLoS ONE. 2014;9(3):e91216. 24614622 10.1371/journal.pone.0091216 PMC3948818

[6] Hegi ME , Diserens AC , Gorlia T , et al. MGMT gene silencing and benefit from temozolomide in glioblastoma. N Engl J Med. 2005;352(10):997‐1003. 15758010 10.1056/NEJMoa043331

[7] Ichimura K , Pearson DM , Kocialkowski S , et al. IDH1 mutations are present in the majority of common adult gliomas but rare in primary glioblastomas. Neuro Oncol. 2009;11(4):341‐347. 19435942 10.1215/15228517-2009-025 PMC2743214

[8] Park C‐K , Park S‐H , Lee S‐H , et al. Methylation status of the MGMT gene promoter fails to predict the clinical outcome of glioblastoma patients treated with ACNU plus cisplatin. Neuropathology. 2009;29(4):443‐449. 19170894 10.1111/j.1440-1789.2008.00998.x

[9] Combs SE , Rieken S , Wick W , et al. Prognostic significance of IDH‐1 and MGMT in patients with glioblastoma: one step forward, and one step back? Radiat Oncol. 2011;6:115. 21910919 10.1186/1748-717X-6-115 PMC3199258

[10] Alves TR , Lima FR , Kahn SA , et al. Glioblastoma cells: a heterogeneous and fatal tumor interacting with the parenchyma. Life Sci. 2011;89(15–16):532‐539. 21641917 10.1016/j.lfs.2011.04.022

[11] Inda MM , Bonavia R , Seoane J . Glioblastoma multiforme: a look inside its heterogeneous nature. Cancers (Basel). 2014;6(1):226‐239. 24473088 10.3390/cancers6010226 PMC3980595

[12] Verhaak R , Hoadley KA , Purdom E , et al. Integrated genomic analysis identifies clinically relevant subtypes of glioblastoma characterized by abnormalities in PDGFRA, IDH1, EGFR, and NF1. Cancer Cell. 2010;17(1):98‐110. 20129251 10.1016/j.ccr.2009.12.020 PMC2818769

[13] Le Mercier M , Fortin S , Mathieu V , Kiss R , Lefranc F . Galectins and gliomas. Brain Pathol. 2010;20(1):17‐27. 19371355 10.1111/j.1750-3639.2009.00270.x PMC2805916

[14] Song L , Tang JW , Owusu L , Sun MZ , Wu J , Zhang J . Galectin‐3 in cancer. Clin Chim Acta. 2014;431:185‐191. 24530298 10.1016/j.cca.2014.01.019

[15] Ilmer M , Mazurek N , Gilcrease MZ , et al. Low expression of galectin‐3 is associated with poor survival in node‐positive breast cancers and mesenchymal phenotype in breast cancer stem cells. Breast Cancer Res. 2016;18(1):97. 27687248 10.1186/s13058-016-0757-6 PMC5043623

[16] Cheng D , Liang B , Li Y . Serum galectin‐3 as a potential marker for gastric cancer. Med Sci Monit. 2015;21:755‐760. 25765552 10.12659/MSM.892386 PMC4370354

[17] Tas F , Bilgin E , Tastekin D , Erturk K , Duranyildiz D . Clinical significance of serum galectin‐3 levels in gastric cancer patients. J Gastrointest Cancer. 2016;47(2):182‐186. 27038444 10.1007/s12029-016-9817-5

[18] Nakamura M , Inufusa H , Adachi T , et al. Involvement of galectin‐3 expression in colorectal cancer progression and metastasis. Int J Oncol. 1999;15(1):143‐148. 10375607 10.3892/ijo.15.1.143

[19] Matsuda Y , Yamagiwa Y , Fukushima K , Ueno Y , Shimosegawa T . Expression of galectin‐3 involved in prognosis of patients with hepatocellular carcinoma. Hepatol Res. 2008;38(11):1098‐1111. 18684128 10.1111/j.1872-034X.2008.00387.x

[20] Ikemori RY , Machado CM , Furuzawa KM , et al. Galectin‐3 up‐regulation in hypoxic and nutrient deprived microenvironments promotes cell survival. PLoS ONE. 2014;9(11):e111592. 25369297 10.1371/journal.pone.0111592 PMC4219723

[21] Park SH , Min HS , Kim B , Myung J , Paek SH . Galectin‐3: a useful biomarker for differential diagnosis of brain tumors. Neuropathology. 2008;28(5):497‐506. 18384511 10.1111/j.1440-1789.2008.00909.x

[22] Silverman AM , Nakata R , Shimada H , Sposto R , DeClerck YA . A galectin‐3‐dependent pathway upregulates interleukin‐6 in the microenvironment of human neuroblastoma. Cancer Res. 2012;72(9):2228‐2238. 22389450 10.1158/0008-5472.CAN-11-2165 PMC3815584

[23] Fukaya Y , Shimada H , Wang LC , Zandi E , DeClerck YA . Identification of galectin‐3‐binding protein as a factor secreted by tumor cells that stimulates interleukin‐6 expression in the bone marrow stroma. J Biol Chem. 2008;283(27):18573‐18581. 18450743 10.1074/jbc.M803115200

[24] Goldman M , Craft B , Kamath A , Brooks AN , Zhu J , Haussler D . The UCSC Xena Platform for cancer genomics data visualization and interpretation. 2018:326470.

[25] Mermel CH , Schumacher SE , Hill B , Meyerson ML , Beroukhim R , Getz G . GISTIC2.0 facilitates sensitive and confident localization of the targets of focal somatic copy‐number alteration in human cancers. Genome Biol. 2011;12(4):R41. 21527027 10.1186/gb-2011-12-4-r41 PMC3218867

[26] van den Bent Mj , Erdem‐Eraslan L , Idbaih A , et al. MGMT‐STP27 methylation status as predictive marker for response to PCV in anaplastic Oligodendrogliomas and Oligoastrocytomas. A report from EORTC study 26951. Clin Cancer Res. 2013;19(19):5513‐5522. 23948976 10.1158/1078-0432.CCR-13-1157

[27] Gravendeel La , Kouwenhoven Mc , Gevaert O , et al. Intrinsic gene expression profiles of gliomas are a better predictor of survival than histology. Cancer Res. 2009;69(23):9065‐9072. 19920198 10.1158/0008-5472.CAN-09-2307

[28] Uhlen M , Fagerberg L , Hallstrom Bm , et al. Tissue‐based map of the human proteome. Science. 2015;347(6220):1260419. 25613900 10.1126/science.1260419

[29] Uhlen M , Oksvold P , Fagerberg L , et al. Towards a knowledge‐based Human Protein Atlas. Nat Biotechnol. 2010;28(12):1248‐1250. 21139605 10.1038/nbt1210-1248

[30] Bresalier RS , Yan PS , Byrd JC , Lotan R , Raz A . Expression of the endogenous galactose‐binding protein galectin‐3 correlates with the malignant potential of tumors in the central nervous system. Cancer. 1997;80(4):776‐787. 9264362

[31] Debray C , Vereecken P , Belot N , et al. Multifaceted role of galectin‐3 on human glioblastoma cell motility. Biochem Biophys Res Commun. 2004;325(4):1393‐1398. 15555581 10.1016/j.bbrc.2004.10.181

[32] Camby I , Belot N , Rorive S , et al. Galectins are differentially expressed in supratentorial pilocytic astrocytomas, astrocytomas, anaplastic astrocytomas and glioblastomas, and significantly modulate tumor astrocyte migration. Brain Pathol. 2001;11(1):12‐26. 11145198 10.1111/j.1750-3639.2001.tb00377.x PMC8098336

[33] Woo JK , Jang J‐E , Kang J‐H , et al. Lectin, galactoside‐binding soluble 3 binding protein promotes 17‐N‐Allylamino‐17‐demethoxygeldanamycin resistance through PI3K/Akt pathway in lung cancer cell line. Mol Cancer Ther. 2017;16(7):1355‐1365. 28336809 10.1158/1535-7163.MCT-16-0574

[34] Piccolo E , Tinari N , Semeraro D , et al. LGALS3BP, lectin galactoside‐binding soluble 3 binding protein, induces vascular endothelial growth factor in human breast cancer cells and promotes angiogenesis. J Mol Med (Berl). 2013;91(1):83‐94. 22864925 10.1007/s00109-012-0936-6

[35] Fogeron M‐L , Müller H , Schade S , et al. LGALS3BP regulates centriole biogenesis and centrosome hypertrophy in cancer cells. Nat Commun. 2013;4:1531. 23443559 10.1038/ncomms2517

[36] Wang Q , Hu B , Hu X , et al. Tumor evolution of glioma‐intrinsic gene expression subtypes associates with immunological changes in the microenvironment. Cancer Cell. 2018;33(1):152. 29316430 10.1016/j.ccell.2017.12.012 PMC5892424

[37] Mahmoud LK , Arfaoui A , Khiari M , et al. Loss of galectin‐3 expression in mucinous colorectal carcinomas is associated with 5'CpG island methylation in Tunisian patients. Appl Immunohistochem Mol Morphol. 2011;19(3):258‐265. 21494181 10.1097/PAI.0b013e3181f869bb

[38] Shui IM , Wong C‐J , Zhao S , et al. Prostate tumor DNA methylation is associated with cigarette smoking and adverse prostate cancer outcomes. Cancer. 2016;122(14):2168‐2177. 27142338 10.1002/cncr.30045 PMC4930391

[39] Hilakivi‐Clarke L , Warri A , Bouker KB , et al. Effects of in utero exposure to ethinyl estradiol on tamoxifen resistance and breast cancer recurrence in a preclinical model. J Natl Cancer Inst. 2017;109(1). 10.1093/jnci/djw188 PMC6255695 27609189

